# Efficient Uranium Removal from Aqueous Solutions Using Silica-Based Adsorbents Functionalized with Various Polyamines

**DOI:** 10.3390/toxics12100704

**Published:** 2024-09-27

**Authors:** Ping Zhang, Hongling Wang, Lifeng Chen, Wenlong Li, Toyohisa Fujita, Shunyan Ning, Yuezhou Wei

**Affiliations:** 1 State Key Laboratory of Featured Metal Materials and Life-Cycle Safety for Composite Structures, School of Resources, Environment and Materials, Guangxi University, Nanning 530004, China; 2115301088@st.gxu.edu.cn (P.Z.); fujitatoyohisa@gxu.edu.cn (T.F.); 2 Institute of Resources Utilization and Rare Earth Development, Guangdong Academy of Sciences, 363 Changxing Road, Guangzhou 510650, China; 3 School of Nuclear Science and Technology, University of South China, 28 Changsheng West Road, Hengyang 421001, China; chenlf@usc.edu.cn (L.C.); liwenlong@usc.edu.cn (W.L.); ningshunyan@usc.edu.cn (S.N.); yzwei@usc.edu.cn (Y.W.); 4 School of Nuclear Science and Engineering, Shanghai Jiao Tong University, 800 Dong Chuan Road, Shanghai 200240, China

**Keywords:** uranium, adsorption, silica, polyamine resins

## Abstract

With the rapid development of nuclear energy, the contamination of environmental water systems by uranium has become a significant threat to human health. To efficiently remove uranium from these systems, three types of silica-based polyamine resins—SiPMA-DETA (SiPMA: silica/poly methyl acrylate; DETA: diethylenetriamine), SiPMA-TETA (TETA: triethylenetetramine), and SiPMA-TEPA (TEPA: tetraethylenepentamine)—were successfully prepared, characterized, and evaluated in batch experiments. Characterization results showed that the silica-based polyamine resins were successfully prepared, and they exhibited a uniform shape and high specific surface area. SiPMA-DETA, SiPMA-TETA, and SiPMA-TEPA had nitrogen contents of 4.08%, 3.72%, and 4.26%, respectively. Batch experiments indicated that these adsorbents could efficiently remove uranium from aqueous solutions with a pH of 5–9. The adsorption kinetics of U(VI) were consistent with the pseudo-second-order model, indicating that the adsorption process was chemisorption and that adsorption equilibrium was achieved within 10 min. SiPMA-TEPA, with the longest polyamine chain, exhibited the highest adsorption capacity (>198.95 mg/g), while SiPMA-DETA, with the shortest polyamine chain, demonstrated the highest U(VI) adsorption efficiency (83%) with 100 mM Na_2_SO_4_. SiPMA-TEPA still removed over 90% of U(VI) from river water and tap water. The spectral analysis revealed that the N-containing functional groups on the ligand were bound to anionic uranium–carbonate species and possibly contributed to the adsorption efficiency. In general, this work presents three effective adsorbents for removing uranium from environmental water systems and thus significantly contributes to the field of environmental protection.

## 1. Introduction

The development of human society cannot be separated from energy, which can be divided into clean and non-clean energy sources. In recent decades, nuclear power, a form of clean energy, has seen significant development [1,2]. Nuclear power plants do not produce pollutants during nuclear power production. However, improper handling of uranium tailing slag during uranium ore mining has resulted in uranium contamination in environmental water systems. Radionuclide concentrations in contaminated areas are up to 200 times those in non-contaminated areas [3]. In addition, radiological experiments with uranium at research institutions, nuclear energy generation, spent fuel treatment, and nuclear weapon manufacturing can lead to uranium contamination [3,4]. These pathways of uranium contamination can result in uranium accumulation in aquatic organisms within environmental water systems [5]. The World Health Organization recommends that drinking water should not contain more than 30 μg/L uranium [6]. Uranium features both metal toxicity and radioactivity, and the ingestion of uranium-contaminated water or fish by humans can cause irreversible damage to the body. Therefore, removing radionuclide uranium from environmental water systems is crucial.

There are various methods for removing radionuclide uranium from solutions, such as membrane filtration [7,8], ion exchange [9,10,11,12], co-precipitation [13,14], and extraction [15]. However, the extraction method has a low treatment efficiency for solutions [15]. As for co-precipitation method, it easily causes secondary pollution. Although the membrane separation method is efficient, its service life is short and it is difficult to regenerate [7,16]. Compared with these separation methods, ion exchange offers advantages in removing uranium from environmental water systems owing to its simplicity, cost-effectiveness, simple operation, and reusability [11].

Adsorbents for uranium removal can be categorized into inorganic, organic, and composite materials [17]. Inorganic adsorbents such as hydroxyapatite [18,19], zero-valent iron [20,21], and natural clinoptilolite zeolite [22,23] have been widely studied. These adsorbents typically enrich uranium through ion exchange, reductive precipitation, or porous physisorption. However, inorganic adsorbents are less selective than organic adsorbents, and carbonates in environmental water systems can affect the adsorption efficiency of inorganic adsorbents [24]. Organic adsorbents, such as metal–organic frameworks (MOFs) [25], covalent organic frameworks [26,27], and ion exchange resins [28,29], offer a more targeted approach. These organic materials usually have a high adsorption capacity, but they also take a long time to reach adsorption equilibrium and are less physically stable in aqueous solutions [4]. MOFs can also be defined as composite materials, but they are also physically unstable in aqueous solutions. In recent years, many researchers have shown great interest in alternative composite materials for uranium removal. For example, silica-based composites are gaining attention owing to the low cost and physical stability of silica spheres [30,31]. In this work, silica spheres and resins are combined to prepare composite materials. In addition, environmental water systems contain many interfering ions. The chemical form of uranium in these systems needs to be considered in the adsorption process using the synthesized silica-based resins.

The chemical form of uranium in environmental water systems is influenced by pH and various coexisting ions. When the pH of environmental water systems is 6.5–8.5, the uranyl ion readily combines with carbonate to form the UO_2_(CO_3_)_2_^2−^ and UO_2_(CO_3_)_3_^4−^ anions [11]. Environmental water systems contain a large number of calcium ions. Consequently, UO_2_(CO_3_)_3_^4−^ readily reacts with Ca^2+^ to form CaUO_2_(CO_3_)_3_, which does not contribute to the adsorption of uranium [4,30]. The concentrations of calcium and magnesium ions vary regionally. Zhang [32] found that in some regions, the calcium ion concentration reached 500 mg/L and was accompanied by sodium, potassium, sulfate, and carbonate ions. Therefore, the designed ion exchange materials still exhibited high uranium adsorption efficiency even in the presence of high concentrations of interfering ions. According to the soft and hard acid–base theory, the uranyl ion is considered a hard acid. Thus, the functional groups of the hard base ligands should contain as many nitrogen or oxygen atoms as possible. Therefore, research into uranium adsorption using polyamine resins has increased in recent years. For example, Cao’s study [33] on uranium removal using PS-N-P resin—prepared by amine-modified P,P-dichlorophenylphosphine—demonstrated a saturated adsorption capacity of 97.60 mg/g and an adsorption efficiency of 99.72% for uranium at pH 5. Amphlett [34] studied the preparation of polyamine-functionalized ion exchange resins for uranium removal and examined how the maximum adsorption capacity varied with the polyamine chain length. The maximum adsorption capacity was 269.50 mg/g, and all resins could extract over 86% of uranium from solutions at pH 1–6. Chen [30] studied the preparation of a novel silica-based anion exchange resin (SAER) for removing uranium from drinking water and found that SAER had a relatively low pressure loss in a packed column and could adsorb uranium with an efficiency of over 80% in a working solution containing 40 mM Na_2_SO_4_. In the studies by Cao and Amphlett [33,34], the solution environment in which the resin adsorbed uranium was relatively simple; however, many interfering ions existed in the environmental water system, which was a challenge for the adsorption of uranium. Chen [30] examined the adsorption efficiency of the resin in real water samples and did not study the relationship between the chain length of the polyamine resin and the adsorption performance. Therefore, in the present study, three types of silica-based polyamine resins with different chain lengths were successfully synthesized. A series of characterizations and evaluations of the adsorption properties of these resins were also completed. The relationship between the polyamine chain length and uranium adsorption efficiency was investigated. Additionally, the adsorption efficiency of the polyamine resin SiPMA-TEPA in environmental water systems was studied. This research provides valuable insights for the synthesis of high-performance silica-based composite resins.

## 2. Experimental Methodology

### 2.1. Materials and Reagents

Silica particles used as carriers for anion exchange resins had an average particle size of 75–150 μm and an average porosity of 69%. Divinylbenzene (DVB) (purity ≥ 80%) was supplied by Shanghai Aladdin Bio-Chem Technology Co., Ltd., Shanghai, China. DVB was stabilized with 1000 mg/L tert-butyl catechol (TBC). The polymerization inhibitor TBC was removed via decompression distillation using a rotary evaporator. Tetraethylenepentamine (TEPA) (purity = 95%), acetophenone, methyl acrylate (MA), diethyl phthalate, UO_2_(NO_3_)•6H_2_O and diethylenetriamine (DETA), all of analytical grade, were purchased from Macklin Biochemical Co., Ltd., Shanghai, China. Triethylenetetramine (TETA) (purity ≥ 70%) was supplied by Shanghai Titan Scientific Co., Ltd., Shanghai, China. Azodiisobutyronitrile (AIBN) of chemical grade was sourced from Tianjin Guangfu Fine Chemical Research Institute (Tianjin, China) and used as an initiator.

### 2.2. Synthesis of SiPMANs

**Figure 1:**
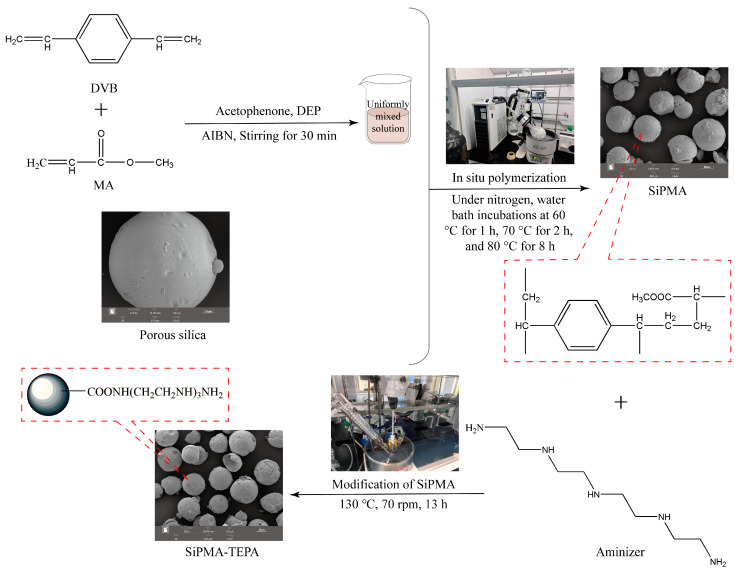
Flowchart for the preparation of SiPMA-TEPA.

The polyamine resins were prepared through an in situ solution polymerization and post-modification strategy, which involved the following two steps: First, SiPMA was synthesized through in situ solution polymerization of DVB and MA within silica spheres. Next, SiPMA was modified with the amine reagents DETA, TETA, and TEPA to produce SiPMA-DETA, SiPMA-TETA, and SiPMA-TEPA polyamine resins, collectively referred to as SiPMANs. The detailed synthesis process is described in Sections S1–S6 of the Supplementary Materials. The schematic of the process for synthesizing SiPMA-TEPA is provided in Figure 1, and the specific chemical reactions are illustrated in Figure S1.

### 2.3. Characterization

The morphology, elemental composition, and distribution of the prepared SiPMANs were analyzed via scanning electron microscopy coupled with energy-dispersive X-ray spectroscopy (SEM-EDS, Tesscan Mira Lms, Brno, Czech Republic). Fourier-transform infrared (FTIR) spectroscopy (IRTracer-100, Shimadzu Corp., Kyoto, Japan) was used to examine the structural information and functional groups of the resin and the changes in chemical bonding at characteristic peaks before and after adsorption. The mass loss of the resin’s organic components was determined using a simultaneous thermal analyzer (TGA-DSC, SDT650, TA Instruments, Newcastle, DE, USA). The pore size distribution, pore volume, and specific surface area of the resin were determined using a Brunauer–Emmett–Teller specific surface area and porosity analyzer (BET, ASAP 2460, Micromeritics, Norcross, GA, USA). The elemental composition was determined using an organic elemental analyzer (Elementar Unicube, Hanau, Germany). Changes in characteristic bonds before and after uranium adsorption on the resin were analyzed via X-ray photoelectron spectroscopy (XPS, Thermo Scientific K-Alpha, Waltham, MA, USA).

### 2.4. Batch Adsorption Experiments

Batch adsorption experiments were conducted to study the adsorption properties of the adsorbent. The effects of the pH level, reaction time, solid-to-liquid ratios, and the presence of various interfering ion species and concentrations on adsorption performance were investigated. First, 0.05 g of SiPMAN adsorbent was weighed and placed into a 40 mL clear glass vial, to which 30 mL of the working solution was added. The mixture was then reacted in a constant-temperature shaker at 25 °C and 120 r/min. After 2 h of shaking, the mixture was filtered through a 13 mm diameter filter with a pore size of 0.45 μm. The concentration of U ions in the filtrate was measured via an inductively coupled plasma atomic emission spectrometer (ICP-AES, Ultima Expert, Paris, France) after dilution with 2.5 *v*/*v*% HNO_3_. The detailed steps of the batch experiment are provided in Section S2 of the Supplementary Materials. SiPMA-TEPA was used to remove uranium from environmental water systems. The environmental water samples were collected from well water (WW) in Mt. Yumu, Xiangjiang River on Jiefang Road, and tap water (TW) from the laboratory. Details are provided in Section S2 and Figure S2 of the Supplementary Materials. The preparation of the SiPMA-TEPA-U sample for the mechanistic analysis is described in Section S5. The adsorption amount (*Q*), adsorption efficiency (*E*), desorption capacity (*Q*_d_), and desorption efficiency (*E*_d_) were used to represent the adsorption performance of the adsorbent. The specific calculation formulas are as follows:

(1) $$Q=\frac{C_{0}-C}{m}\times V$$

(2) $$E=\frac{C_{0}-C}{C_{0}}\times 100\%$$

(3) $$Q_{d}=C_{d}\times \frac{V}{m}$$

(4) $$E_{d}=\frac{Q_{d}}{Q}\times 100\%$$

where *m* (g) is the mass of the adsorbent; *V* (mL) is the volume of the liquid phase; *C*_0_ and *C* (mg/L) represent the ion concentrations in the liquid phase before and after adsorption by the adsorbent, respectively; and *C*_d_ (mg/L) is the ion concentration in the liquid phase after desorption. Additionally, the solid-to-liquid ratio during the elution process is the same as that during the adsorption process.

## 3. Results and Discussion

### 3.1. Material Characterization

#### 3.1.1. SEM–EDS Analysis

**Figure 2:**
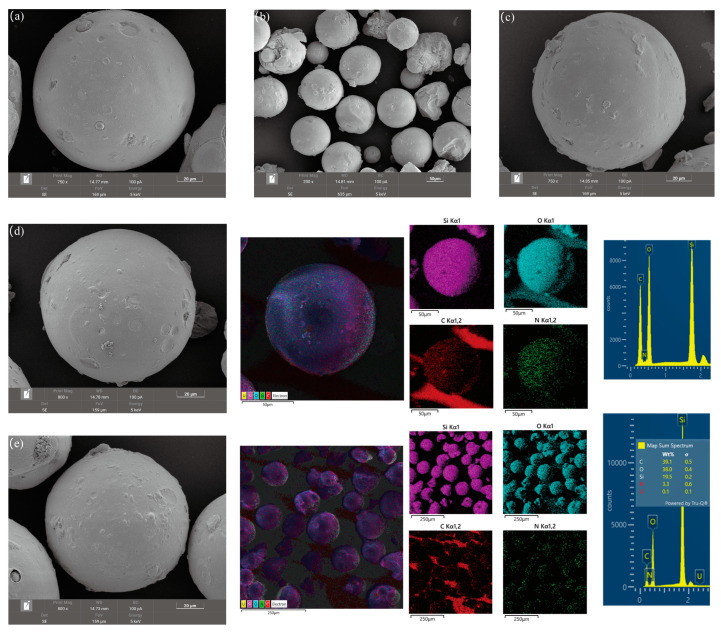
SEM–EDS images of the surfaces of SiO_2_ (**a**,**b**), SiPMA (**c**), SiPMA-TEPA (**d**), and SiPMA-TEPA-U (**e**).

The electron microscopic morphology of the SiO_2_ carrier (75–150 μm, Figure 2a,b) revealed that it exhibited a high degree of sphericity and a smooth spherical surface. The SiPMA surface was also smooth, as shown in Figure 2c. The morphology of the SiPMA-TEPA adsorbent is depicted in Figure 2d; the surface of SiPMA-TEPA appeared slightly rougher than that of SiPMA.

The elemental distribution results, as shown on the right side of the energy spectra in Figure 2d,e, and the elemental analysis results (Table S1) indicated that the elements C, N, O, and U were evenly distributed within the silica particles. This suggests that SiPMA-TEPA successfully adsorbed uranium. These findings confirm the successful preparation of the silica-based polyamine composite resin.

#### 3.1.2. TG–DSC Analysis

**Figure 3:**
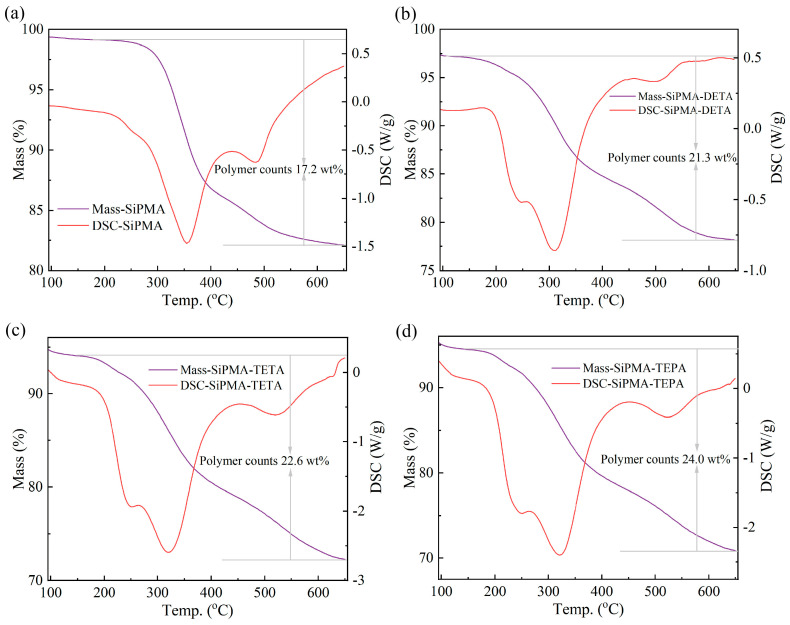
TG–DSC analysis of SiPMA (**a**), SiPMA-DETA (**b**), SiPMA-TETA (**c**), and SiPMA-TEPA (**d**).

To determine the organic contents of SiPMA and SiPMANs, a thermogravimetric analysis was conducted between 25 °C and 650 °C. The DSC curves revealed that the peak decomposition temperatures for SiPMA, SiPMA-DETA, SiPMA-TETA, and SiPMA-TEPA were approximately 354 °C, 311 °C, 320 °C, and 321 °C, respectively (Figure 3a–d), indicating the decomposition temperatures of the organic polymers [30]. The mass losses for SiPMA-DETA, SiPMA-TETA, and SiPMA-TEPA were 21.3%, 22.6%, and 24%; this indicates that 21.3%, 22.6%, and 24% of the resins were incorporated into the silica spheres, respectively.

#### 3.1.3. BET Analysis

**Figure 4:**
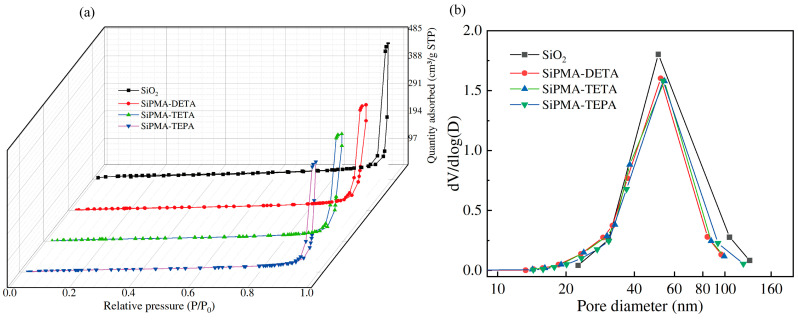
N_2_-adsorption–desorption isotherms (**a**) and pore size distributions (**b**) of SiO_2_ and SiPMANs.

To elucidate the pore size distribution and specific surface area of SiPMANs, a BET analysis was performed. The N_2_ adsorption–desorption isotherms of SiPMANs are shown in Figure 4a. The presence of a type IV hysteresis loop in the adsorption curve indicates that SiPMANs had a mesoporous structure [35]. The pore size of SiPMA-TEPA did not differ significantly from those of SiPMA-TETA and SiPMA-DETA, presumably because the polymerization of organic monomers did not fully penetrate the pore channels of SiO_2_. However, SiPMA-TEPA still exhibited excellent adsorption performance. As shown in Table 1, the specific surface area, pore volume, and pore diameter of SiPMANs were all lower than those of SiO_2_. This indicates that the organic polymer occupied space within the SiO_2_ pores.

#### 3.1.4. FTIR Analysis

**Figure 5:**
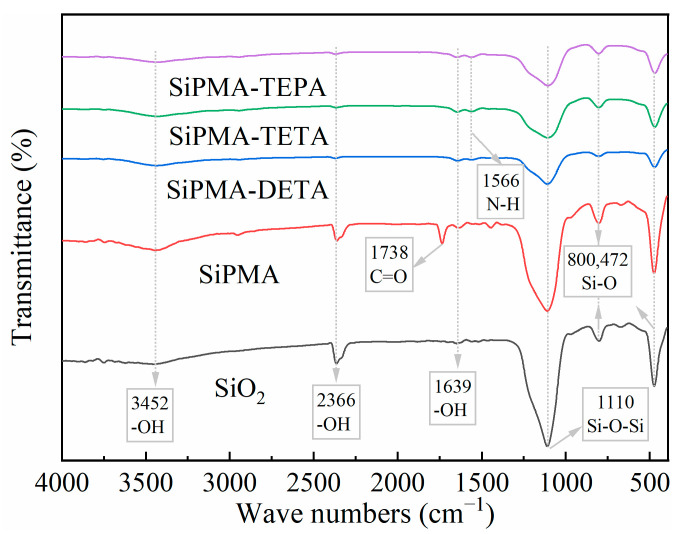
FT-IR spectra of SiO_2_, SiPMA, and SiPMANs.

The FTIR spectra of SiO_2_, SiPMA, and SiPMANs are shown in Figure 5. The peak at 3452 cm^−1^ corresponded to the stretching vibration of the adsorbed aqueous hydroxyl groups. The peaks at 1639 cm^−1^ and 2366 cm^−1^ corresponded to the bending vibrations of these hydroxyl groups [36,37]. The peaks at 1738 cm^−1^ corresponded to the vibration of the carbonyl group in the intermediate methyl acrylate [30]. The peaks at 472 cm^−1^, 800 cm^−1^, and 1110 cm^−1^ corresponded to SiO_2_ [30,37]. The spectra of all of the polyamine resins exhibited these characteristic peaks, which indicated that the structure of the synthesized adsorbent was stabilized. The spectrum of SiPMANs featured a unique peak at 1566 cm^−1^, which corresponded to the bending vibration of N-H [30]. These results demonstrated that the surface of the SiPMA material was successfully amidated, and SiPMANs were successfully prepared.

### 3.2. Batch Adsorption Experiments

**Figure 6:**
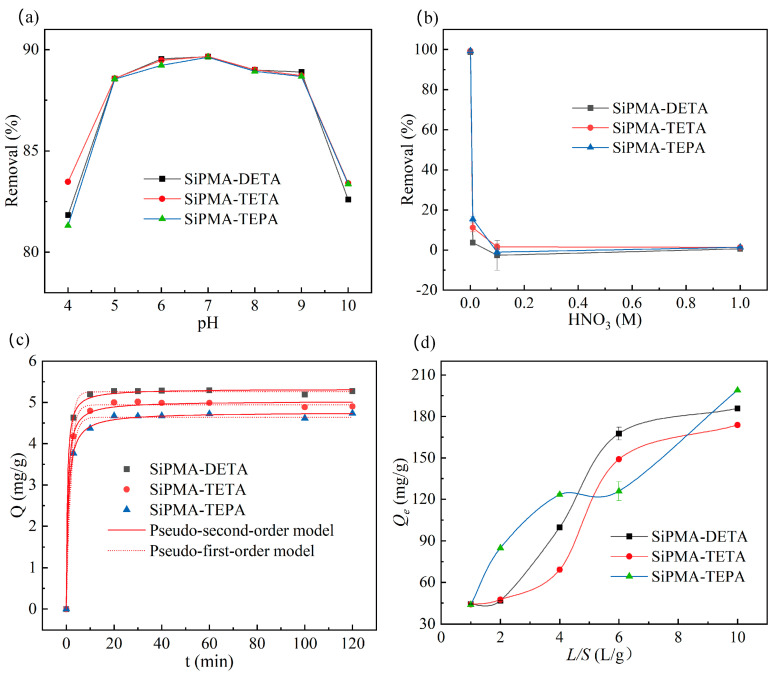
Effect of pH on the uranium removal efficiency (**a**); effects of the nitric acid concentration on the uranium removal efficiency (**b**); plots of the pseudo-first-order and pseudo-second-order kinetic models for uranium adsorption (**c**); and amount of uranium adsorbed as a function of the liquid-to-solid ratio (**d**) (C_0_(NaHCO_3_) = 3 mmol/L, initial pH 7 ± 0.05, (**a**) C_0_(U) ≈ 1 mg/L, *t* = 24 h, *m*/*v*: 0.05 g/30 mL; (**b**) C_0_(U) ≈ 10 mg/L, *t* = 2 h; (**c**) C_0_(U) ≈ 10 mg/L; and (**d**) C_0_(U) ≈ 50 mg/L, *t* = 2 h).

As shown in Figure 6a, all three polyamine adsorbents were highly effective in removing uranium from aqueous solutions at pH 4–10, with particularly high efficiency at pH 5–9, where the removal efficiency remained nearly constant at ~90%. As the H^+^ concentration in the solution increased, the nitrogen-containing functional groups on the polyamine resin ligands became more easily protonated. However, the chemical form of uranium gradually converted from UO_2_ (CO_3_)_3_^4−^ to UO_2_^2+^ [30], which is not conducive to ion exchange. This resulted in a decrease in adsorption efficiency, particularly at pH 4, where the decrease was most pronounced. As shown in Figure 6b, the polyamine adsorbents hardly adsorbed uranium when the HNO_3_ concentration exceeded 0.1 M. As fewer H^+^ ions were present in the solution, the nitrogen-containing functional groups on the polyamine resin ligands were less likely to be protonated, which hindered ion exchange. Consequently, the adsorption efficiency of the polyamine resins for uranium decreased, especially at pH 10. The pH of environmental water systems varies owing to regional differences, with an overall range of 6–8 [30]. This indicates that SiPMANs are widely applicable to diverse environmental water conditions.

The adsorption equilibrium time is a crucial performance indicator for ion exchange resins. Figure 6c illustrates the effect of the reaction time on uranium adsorption. Adsorption rapidly increased within the first 5 min, and equilibrium was reached in less than 10 min. This rapid equilibrium was due to the SiPMAN resins having a larger specific surface area and pore size, which facilitated the entry of uranium.

The experimental data were fitted using the pseudo-first-order kinetic model (Equation (S1)) [38] and the pseudo-second-order kinetic model (Equation (S2)) [36] to investigate the adsorption mechanism. The fitting results are shown in Table 2.

According to the *R*^2^ values in Table 2, the pseudo-second-order kinetic model was more suitable for describing the adsorption mechanism than the pseudo-first-order kinetic model, indicating that the adsorption of uranium onto SiPMA-TEPA was chemisorptive [39].

The saturated adsorption capacity of a resin is crucial for its practical application. This capacity is generally determined from the adsorption isotherm. However, at pH levels close to 7, highly concentrated uranium can hydrolyze and precipitate, which can complicate the measurement of the adsorption capacity [40]. Thus, a solid-to-liquid ratio experiment was conducted to investigate the maximum adsorption capacity of the resin.

As shown in Figure 6d, the maximum adsorption capacity of the SiPMA-TEPA adsorbent exceeded 198.95 mg/g. The SiPMA-TEPA adsorbent with the longest polyamine chain exhibited the highest adsorption capacity, consistent with the findings of Amphlett’s study [34]. The adsorption capacity of the SAER adsorbent with the same polyamine group was 124 mg/g [30,40]. Adsorption isotherm experiments were conducted to elucidate the uranium adsorption mechanism of SiPMA-TEPA. The detailed experimental procedures are described in Section S2 of the Supplementary Materials. The saturation U(VI)-adsorption capacity of SiPMA-TEPA is illustrated in Figure S3. The fitting results of the Langmuir model (Equation (S3)) [12] and the Freundlich model (Equation (S4)) [41] are shown in Table S2. The *R*^2^ value for the Langmuir model was higher than that for the Freundlich model, indicating that the adsorption process aligned with the Langmuir model, which represents a monolayer chemisorption process.

**Figure 7:**
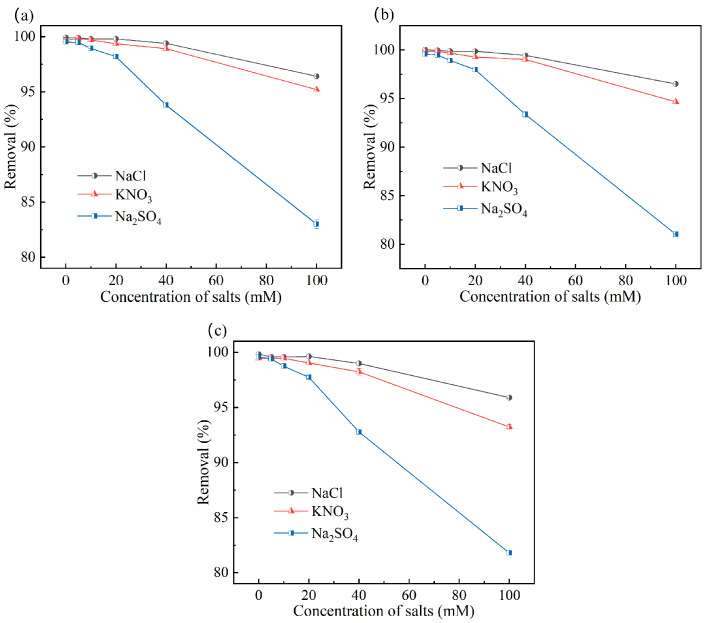
Effects of specific salts on the uranium removal efficiencies of SiPMA-DETA (**a**), SiPMA-TETA (**b**), and SiPMA-TEPA (**c**) (C_0_(U) ≈ 10 mg/L, C_0_(NaHCO_3_) = 3 mmol/L; *t* = 2 h; *m*/*v*: 0.05 g/30 mL).

Interfering ions in environmental water predominantly include anions such as Cl^−^, NO_3_^−^, and SO_4_^2−^ and cations such as Na^+^, K^+^, Ca^2+^, and Mg^2+^. To compare the adsorption selectivity of the three polyamine resins, experiments were designed using salts with concentrations ranging from 0 mM to 100 mM. The three types of polyamine resins were reacted in these three salts (NaCl, KNO_3_, and Na_2_SO_4_) with different concentrations. The results are shown in Figure 7. As the number of interfering ions in the solution increased, the interfering ions competed with UO_2_(CO_3_)_2_^2−^ or UO_2_(CO_3_)_3_^4−^ for adsorption with the protonated polyamine resin.

The adsorption selectivity of the three polyamine resins for uranium remained high at a 100 mM salt concentration. Specifically, the uranium adsorption efficiency of all three polyamine adsorbents exceeded 90% in the presence of 100 mM NaCl or KNO_3_ and surpassed 80% in the presence of 100 mM Na_2_SO_4_. SiPMA-DETA, with the shortest polyamine chain, exhibited a higher selectivity for uranium than the other two polyamine resins. The uranium adsorption efficiency of SiPMA-DETA reached 83% in 100 mM Na_2_SO_4_, likely owing to the relatively higher content of primary amines in SiPMA-DETA, which enhanced selectivity [34].

**Figure 8:**
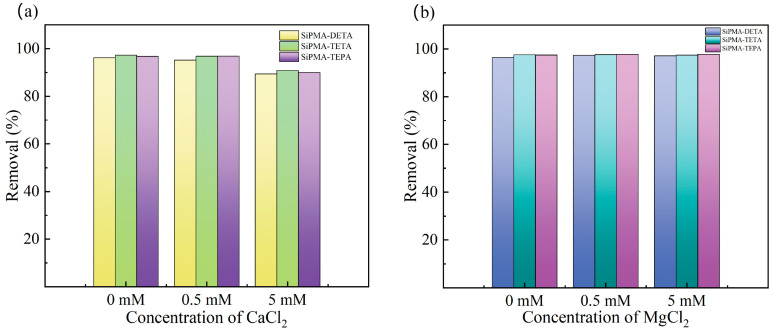
Effects of Ca^2+^ (**a**) and Mg^2+^ (**b**) on the uranium removal efficiency (C_0_(U) ≈ 1 mg/L, C_0_(NaHCO_3_) = 3 mmol/L; *t* = 2 h; *m*/*v*: 0.05 g/30 mL).

As shown in Figure 8, the adsorption efficiencies of the three polyamine resins were almost unchanged with an increase in the MgCl_2_ concentration to 5 mM, while the removal efficiencies of the three resins decreased with an increase in the CaCl_2_ concentration to 5 mM, which corresponded to the average concentration of Ca^2+^ in groundwater. The decrease in the removal efficiency of the resins was due to the tendency of Ca^2+^ to combine with UO_2_(CO_3_)_3_^4−^ to produce a neutral molecule, Ca_2_UO_2_(CO_3_)_3_ [30,42]. The removal efficiency of uranium remained around 90% in 5 mM CaCl_2_ or MgCl_2_, demonstrating the practical application potential of SiPMANs. In summary, the SiPMA-TEPA resin demonstrated superior overall adsorption performance and was selected for further study.

### 3.3. Batch Desorption Experiments and Reusability

**Figure 9:**
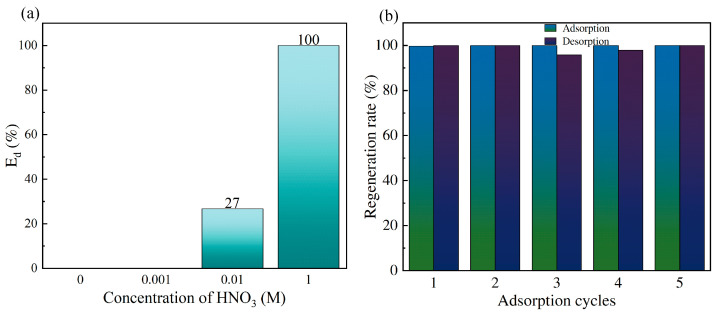
Uranium desorption efficiency with different eluents (**a**) and (**b**) reproducibility of SiPMA-TEPA ((**a**) *C*_0_(U) ≈ 1 ppm, pH = 7, *m*/*v*: 0.05 g/10 mL).

The uranium adsorbed by SiPMA-TEPA was desorbed using different concentrations of nitric acid. As shown in Figure 9a, the resin achieved complete desorption of uranium with 1 M nitric acid. Additionally, the uranyl ions were completely desorbed from the resin in less than 3 min (Figure S4). Reusability and stability evaluations of SiPMA-TEPA were conducted using 1 M nitric acid as the desorbent. As shown in Figure 9b, the adsorbent’s adsorption and desorption efficiencies remained above 95% after five cycles, demonstrating good stability and reusability.

### 3.4. Evaluation of the Uranium Removal Performance in Environmental Water Systems

**Figure 10:**
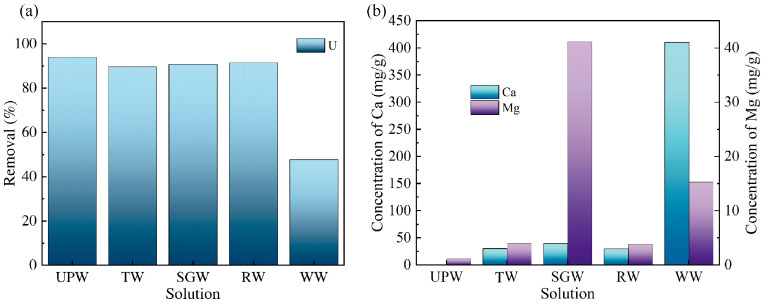
Uranium removal by SiPMA-TEPA in different water systems (**a**) and (**b**) Ca^2+^, Mg^2+^ ion concentrations in different water systems (temperature: 298 K, *C*_0_(U) ≈ 1 ppm, t: 2 h, *m*/*v*: 0.05 g/30 mL).

The results for the uranium removal efficiency of SiPMA-TEPA in environmental water systems are shown in Figure 10a. SiPMA-TEPA achieved the highest removal rate of 94.03% for uranium in ultrapure water (UPW). Uranium removal exceeded 90% in TW, river water (RW), and simulated groundwater (SGW). The configuration of the simulated water samples was based on the literature [43,44]. Details are provided in Section S2 of the Supplementary Materials and Figure S2. The uranium removal efficiency was lowest in WW, owing to the high concentration of Ca^2+^ (410.4 ppm) in the groundwater environment, which was significantly higher than that in other water systems. The elevated Ca^2+^ concentration led to the formation of the neutral molecule Ca_2_(UO_2_)(CO_3_)_3_ and thus inhibited the resin’s adsorption of uranium. Nevertheless, the excellent uranium removal efficiency observed in TW and RW indicated that SiPMA-TEPA was suitable for practical applications.

### 3.5. Adsorption Mechanism Study

#### 3.5.1. FTIR and XPS Analyses

**Figure 11:**
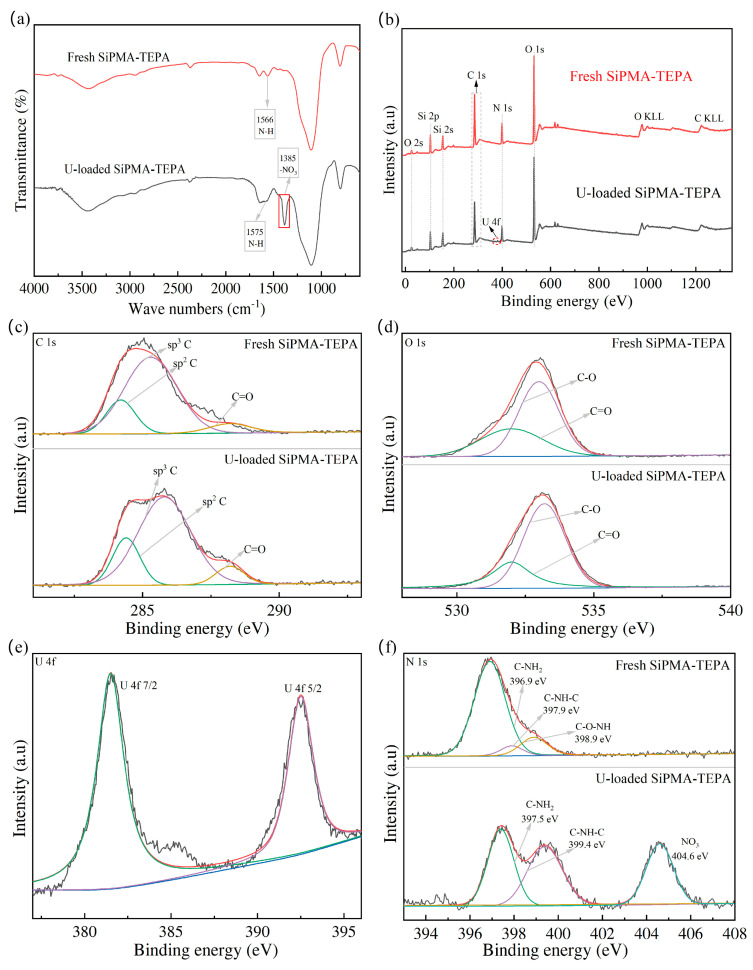
(**a**) FTIR spectra of SiPMA-TEPA and SiPMA-TEPA loaded with U; (**b**) XPS spectra of SiPMA-TEPA and SiPMA-TEPA loaded with U, including fine XPS C 1s and O 1s spectra (**c**,**d**); (**e**) fine XPS U 4f spectrum; and (**f**) fine XPS N 1s spectrum.

To further study the mechanism of U(VI) adsorption by SiPMA-TEPA, FTIR and XPS analyses were performed. As shown in Figure 11a, the characteristic peak at 1566 cm^−1^ for SiPMA-TEPA corresponded to the bending vibration of the N-H bond of the polyamine functional group of the resin [30]; moreover, the peak of the N-H bond of the resin SiPMA-TEPA-U shifted, and a new peak for nitrate appeared. This indicates that the polyamine functional groups in the resin were involved in coordination. A peak for nitrate [45] (1385 cm^−1^) was detected on the adsorbent after uranium adsorption; this peak corresponded to a small amount of nitric acid extracted by SiPMA-TEPA.

**Figure 12:**
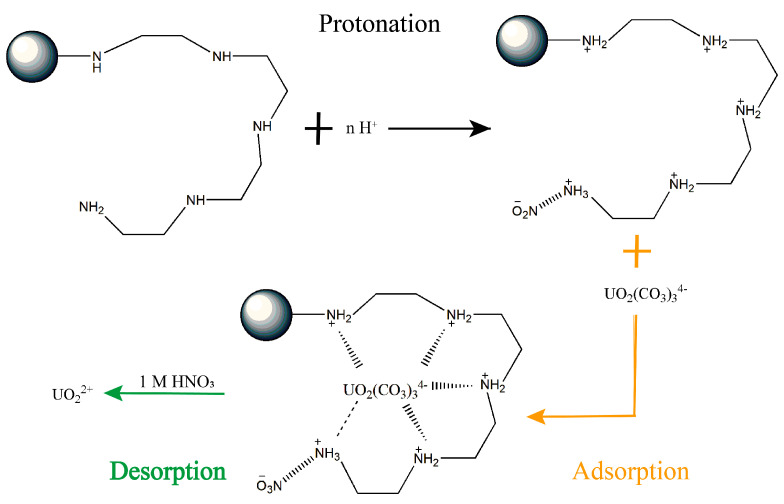
Schematic of the adsorption and desorption mechanisms of uranium from environmental water using the SiPMA−TEPA adsorbent.

The full XPS spectra of the SiPMA-TEPA adsorbent before and after adsorption of U(VI) are shown in Figure 11b. The appearance of the characteristic U(VI) peak on the U-loaded SiPMA-TEPA adsorbent indicates that U(VI) was successfully adsorbed. Figure 11c shows the XPS fine scan C 1s spectrum. The peak area percentage of C=O (288.5 eV) [46,47,48] became larger after uranium adsorption by SiPMA-TEPA, indicating the contribution of carbonate. Figure 11d presents the high-resolution XPS O 1s spectrum. Changes in the peak areas of C=O (532 eV) and C-O (533 eV) [49,50] after uranium adsorption further confirmed that CO_3_^2−^ was bound to UO_2_^2+^. Figure 11c illustrates the high-resolution U 4f spectrum, where the peaks at 381.5 eV and 392.5 eV correspond to the 7/2 and 5/2 U 4f levels, respectively. Figure 11f shows the high-resolution XPS N 1s spectrum. The binding energy of N-H shifted from 396.9 eV to 397.5 eV, and the binding energy of N-C shifted from 397.9 eV to 399.4 eV. After U adsorption, the resin showed a characteristic peak between nitrate and uranium at 404.6 eV [38]; this indicates that the primary amine groups of the resin extracted nitric acid, further corroborating the FTIR results. Additionally, the binding energy of N-C shifted from 397.9 eV to 399.4 eV, suggesting the involvement of N-containing functional groups on the polymer in the coordination [30,51]. The predominant chemical form of uranium at pH 6–7 was UO_2_(CO_3_)_3_^4−^, and all the polyamine groups on the adsorbent were protonated to coordinate with UO_2_(CO_3_)_3_^4−^. The mechanism is illustrated in Figure 12.

#### 3.5.2. Comparison of SiPMA-TEPA with Other Materials

Furthermore, the SiPMA-TEPA adsorbent was compared with other materials for the treatment of uranium (Table 3). The results showed that SiPMA-TEPA had a relatively high adsorption capacity and an extremely fast adsorption equilibrium time. SiPMA-TEPA exhibited excellent adsorption properties.

## 4. Conclusions

Three novel silica-based polyamine composite resins were synthesized through in situ solution polymerization and post-modification strategies. Among them, the resin with the longest polyamine chain, TEPA, demonstrated the best overall performance. SiPMA-DETA and SiPMA-TETA were mesoporous adsorbents, while SiPMA-TEPA was a macroporous adsorbent. Adsorption equilibrium was achieved in less than 10 min, and desorption was completed in under 3 min. SiPMA-DETA, with the shortest polyamine chain, exhibited the highest uranium adsorption efficiency of 83% in 100 mM Na_2_SO_4_, and SiPMA-TEPA exhibited the highest adsorption capacity (>198.95 mg/g). The adsorption process for SiPMA-TEPA followed the pseudo-second-order kinetic model and the Langmuir isotherm model, indicating a chemisorption process. Additionally, SiPMA-TEPA removed over 90% of uranium from both TW and RW. After five cycles of adsorption and desorption, the removal efficiency remained high, demonstrating excellent stability. FTIR and XPS analyses revealed that the N-containing functional groups in the SiPMA-TEPA adsorbent bound to the UO_2_(CO_3_)_3_^2−^ anion in the solution and acted as an effective adsorption site for the UO_2_(CO_3_)_3_^2−^ anion in the solution. Overall, SiPMA-TEPA demonstrated strong potential for the rapid treatment of uranium-containing water in environmental water systems.

## Figures and Tables

**Table 1:** BET results for SiO_2_ and SiPMANs obtained via nitrogen adsorption.

Materials	BET Surface Area (m^2^/g)	Pore Volume (cm^3^/g)	Pore Size (nm)
SiO_2_	74.59	0.76	56.08
SiPMA-DETA	48.38	0.25	23.05
SiPMA-TETA	46.86	0.59	23.77
SiPMA-TEPA	46.42	0.61	52.56

**Table 2:** Parameters obtained by fitting the data with the pseudo-first-order and pseudo-second-order models.

Adsorbents	Pseudo-First-Order Kinetic Model			Pseudo-Second-Order Kinetic Model			*Q*_e,exp_ (mg/g)
	*K*_1_	*Q*_e_	*R* ^2^	*K*_2_	*Q*_e_	*R* ^2^	
SiPMA-DETA	0.71	5.26	0.999	0.45	5.33	0.999	5.26
SiPMA-TETA	0.62	4.94	0.998	0.34	5.03	0.997	4.962
SiPMA-TEPA	0.55	4.64	0.995	0.27	4.76	0.999	4.681

**Table 3:** Comparison of the adsorption performances of SiPMA-TEPA and various solid materials for uranium removal.

Adsorbent	*Q*_max_ (mg/g)	*t*_e_ (min)	Temperature (K)	Reference
SBA/EnSA	105.3	1675	298	[52]
MHO	106.4	-	293	[53]
FMHO	133.3	480	293	[53]
SAER	124	8	298	[30]
PS-N-P	94.95	240	298	[33]
SiPMA-TEPA	270	10	298	This work
Sal-APS-MCM-41	10	<1	298	[54]
M-Ti_2_CTx	470	2880	298	[55]
PEI/GO	145.1	90	303	[56]

## Data Availability

Data are contained within the article and Supplementary Materials.

## Supplementary Materials

The following supporting information can be downloaded at https://www.mdpi.com/article/10.3390/toxics12100704/s1: Section S1. Synthesis of SiPMANs; Section S2. Batch experiments; Section S3. Introduction of the mathematical models used; Section S4. Experiments on the reusability of SiPMA-TEPA; Section S5. Sample preparation for the adsorption mechanism investigation; Section S6. Experimental results; Figure S1. MA-and-DVB-crosslinked copolymerization (a) and substitution reaction between SiPMA and TEPA (b); Table S1. Elemental contents of SiPMAN resins; Table S2. Parameters obtained by fitting adsorption isotherm data for different solutions; Figure S2. Well water of Mt. Yumu, Hengyang City (a); water collected from Xiangjiang River, Jiefang Road, Hengyang City (b); and tap water from Science and Innovation Building 406, University of South China (c); Figure S3. Adsorption isotherms for uranium removal by SiPMA-TEPA in ultrapure water (UPW) and groundwater (temperature 298 K, t: 2 h, pH = 6, *m*/*v*: 0.01 g/30 mL); Figure S4. Desorption kinetics.

## References

[1] Ayare S.D., Doltade S., Tekade S. (2024). A review on current scenario of energy, nuclear reactor technology and cold trap. Clean Technol. Environ. Policy.

[2] Rogalev N., Rogalev A., Kindra V., Zlyvko O., Osipov S. (2023). An Overview of Small Nuclear Power Plants for Clean Energy Production: Comparative Analysis of Distributed Generation Technologies and Future Perspectives. Energies.

[3] Marovic G., Sencar J., Bronzovic M., Franic Z., Kovac J. (2006). Radioactive waste due to electric power and mineral fertiliser production. Arh. Za Hig. Rada I Toksikol..

[4] Xie Y., Chen C.L., Ren X.M., Wang X.X., Wang H.Y., Wang X.K. (2019). Emerging natural and tailored materials for uranium-contaminated water treatment and environmental remediation. Prog. Mater. Sci..

[5] Bergmann M., Graca M.A.S. (2020). Bioaccumulation and Dispersion of Uranium by Freshwater Organisms. Arch. Environ. Contam. Toxicol..

[6] Cotruvo J.A. (2017). 2017 WHO Guidelines for Drinking Water Quality: First Addendum to the Fourth Edition. J. Am. Water Work. Assoc..

[7] Zhou H., Leng J., Guo Y., Liu J., Ge K., Zhang W. (2022). Research progress of membrane technology in treatment of uranium-containing wastewater from nuclear industry. Ind. Water Treat..

[8] Yan B.J., Ma C.X., Gao J.X., Yuan Y.H., Wang N. (2020). An Ion-Crosslinked Supramolecular Hydrogel for Ultrahigh and Fast Uranium Recovery from Seawater. Adv. Mater..

[9] Chen F., Lv M., Ye Y., Miao S., Tang X., Liu Y., Liang B., Qin Z., Chen Y., He Z. (2022). Insights on uranium removal by ion exchange columns: The deactivation mechanisms, and an overlooked biological pathway. Chem. Eng. J..

[10] Wang L., Badr H.O., Yang Y., Cope J.H., Ma E.Z., Ouyang J.F., Yuan L.Y., Li Z.J., Liu Z.R., Barsoum M.W. (2023). Unique hierarchical structures of one dimensional lepidocrocite titanate with cation-exchangeable sites for extraordinary selective actinide capture for water purification. Chem. Eng. J..

[11] Chen L.F., Ning S.Y., Huang Y.L., Chen Y.L., Ju Z.P., He X.W., Lu L.Y., Zhou H.L., Wang X.P., Wu Y. (2020). Effects of speciation on uranium removal efficiencies with polyamine-functionalized silica composite adsorbent in groundwater. J. Clean. Prod..

[12] Chen L., Yin X., Yu Q., Lu S., Meng F., Ning S., Wang X., Wei Y. (2019). Rapid and selective capture of perrhenate anion from simulated groundwater by a mesoporous silica-supported anion exchanger. Microporous Mesoporous Mater..

[13] Wang R.X., Li M.Z., Liu T., Li X.Y., Zhou L., Tang L., Gong C.Y., Gong X., Yu K.F., Li N. (2022). Encapsulating carbon-coated nano zero-valent iron particles with biomass-derived carbon aerogel for efficient uranium extraction from uranium-containing wastewater. J. Clean. Prod..

[14] Jun B.M., Kim H.H., Rho H., Seo J., Jeon J.W., Nam S.N., Park C.M., Yoon Y. (2023). Recovery of rare-earth and radioactive elements from contaminated water through precipitation: A review. Chem. Eng. J..

[15] Dawn S.S., Vishwakarma V. (2021). Recovery and recycle of wastewater contaminated with heavy metals using adsorbents incorporated from waste resources and nanomaterials-A review. Chemosphere.

[16] Yu Y., Liu J., Chen S., Song Y., Chen R., Yu J., Zhu J., Li Y., Liu Q., Wang J. (2024). Bioinspired electrostatic layer-by-layer assembly membranes constructed based on mild strategy for uranium extraction from seawater. Chem. Eng. J..

[17] Dinis M.d.L., Fiuza A. (2021). Mitigation of Uranium Mining Impacts-A Review on Groundwater Remediation Technologies. Geosciences.

[18] Huang H., Qiang L., Fan M., Liu Y., Yang A., Chang D., Li J., Sun T., Wang Y., Guo R. (2024). 3D-printed tri-element-doped hydroxyapatite/polycaprolactone composite scaffolds with antibacterial potential for osteosarcoma therapy and bone regeneration. Bioact. Mater..

[19] Kong L.J., Ruan Y., Zheng Q.Y., Su M.H., Diao Z.H., Chen D.Y., Hou L.A., Chang X.Y., Shih K.M. (2020). Uranium extraction using hydroxyapatite recovered from phosphorus containing wastewater. J. Hazard. Mater..

[20] Tang C., Wang X., Zhang Y., Liu N., Hu X. (2024). Corrosion behaviors and kinetics of nanoscale zero-valent iron in water: A review. J. Environ. Sci..

[21] Zhu F., Lu H., Li T., He S., Xu H. (2024). Degradation on BDE209 in the soil by kaolin-supported sulfurized nano-zero-valent iron activated persulfate system: Insights mechanism and DFT calculations. J. Environ. Chem. Eng..

[22] Velarde L., Nabavi M.S., Escalera E., Antti M.-L., Akhtar F. (2023). Adsorption of heavy metals on natural zeolites: A review. Chemosphere.

[23] Yang T.Z., Zhang W.M., Liu H.Y., Guo Y.D. (2020). Enhanced removal of U(VI) from aqueous solution by chitosan-modified zeolite. J. Radioanal. Nucl. Chem..

[24] Katsoyiannis I.A., Zouboulis A.I. (2013). Removal of uranium from contaminated drinking water: A mini review of available treatment methods. Desalin. Water. Treat..

[25] Ma L., Huang C., Yao Y.Y., Fu M.T., Han F., Li Q.N., Wu M.H., Zhang H.J., Xu L., Ma H.J. (2023). Self-assembled MOF microspheres with hierarchical porous structure for efficient uranium adsorption. Sep. Purif. Technol..

[26] Zhong X., Lu Z.P., Liang W., Hu B.W. (2020). The magnetic covalent organic framework as a platform for high-performance extraction of Cr(VI) and bisphenol a from aqueous solution. J. Hazard. Mater..

[27] Mei D.C., Liu L.J., Yan B. (2023). Adsorption of uranium (VI) by metal-organic frameworks and covalent-organic frameworks from water. Coord. Chem. Rev..

[28] Elzoghby A.A. (2021). Kinetic and equilibrium studies for U(VI) and Cd(II) sorption from commercial phosphoric acid using C100H resin. J. Radioanal. Nucl. Chem..

[29] Lee H.-K., Park W., Chang S., Jeon H., Park S. (2022). Uranium Recovery from Sulfate-Based Acidic Soil Washing Effluent Using Ion-Exchange Resins. Water Air Soil Pollut..

[30] Chen L., Chen Y., Wang X., Wei Y., He L., Tang F. (2017). A novel silica-based anion exchange resin used for removing uranium from drinking water. J. Radioanal. Nucl. Chem..

[31] Chen L., Zhang J., Huang Y., Peng C., Chen Y., Lu L., Wang X., Wei Y. (2021). An anion exchange pretreatment method for the determination of low-level uranium in the environmental water samples. J. Environ. Radioact..

[32] Zhang Z., Shao C., Wen X., He Z. (2010). Simultaneous Determination of Calcium and Magnesium in Groundwater Samples by Ion Chromatography. Rock Miner. Anal..

[33] Cao Q., Liu Y., Kong X., Zhou L., Guo H. (2013). Synthesis of phosphorus-modified poly(styrene-co-divinylbenzene) chelating resin and its adsorption properties of uranium(VI). J. Radioanal. Nucl. Chem..

[34] Amphlett J.T.M., Ogden M.D., Foster R.I., Syna N., Soldenhoff K., Sharrad C.A. (2018). Polyamine functionalised ion exchange resins: Synthesis, characterisation and uranyl uptake. Chem. Eng. J..

[35] Zhang W., Ning S., Zhang S., Wang S., Zhou J., Wang X., Wei Y. (2019). Synthesis of functional silica composite resin for the selective separation of zirconium from scandium. Microporous Mesoporous Mater..

[36] Liu H., Ning S., Li Z., Zhang S., Chen L., Yin X., Fujita T., Wei Y. (2022). Preparation of a novel silica-based N-donor ligand functional adsorbent for efficient separation of palladium from high level liquid waste. Sep. Purif. Technol..

[37] Zhang S., Ning S., Liu H., Wang X., Wei Y., Yin X. (2021). Preparation of ion-exchange resin via in-situ polymerization for highly selective separation and continuous removal of palladium from electroplating wastewater. Sep. Purif. Technol..

[38] Ning S., Zhang S., Zhang W., Zhou J., Wang S., Wang X., Wei Y. (2020). Separation and recovery of Rh, Ru and Pd from nitrate solution with a silica based IsoBu-BTP/SiO_2_-P adsorbent. Hydrometallurgy.

[39] Tang J., Liao L.Y., He X.X., Lv L.D., Yin X.B., Li W.L., Wei Y.Z., Ning S.Y., Chen L.F. (2024). Efficient separation of radium from natural thorium using a mesoporous silica-supported composite resin with sulfonic acid groups for the acquisition of targeted α-nuclides ^212^Pb. Chem. Eng. J..

[40] Li K., Xiong T., Liao J., Lei Y., Zhang Y., Zhu W. (2022). Design of MXene/graphene oxide nanocomposites with micro-wrinkle structure for efficient separating of uranium(VI) from wastewater. Chem. Eng. J..

[41] Zhang W., Yu S., Zhang S., Zhou J., Ning S., Wang X., Wei Y. (2019). Separation of scandium from the other rare earth elements with a novel macro-porous silica-polymer based adsorbent HDEHP/SiO_2_-P. Hydrometallurgy.

[42] Wei Y.Z., Kumagai M., Takashima Y., Asou M., Namba T., Suzuki K., Maekawa A., Ohe S. (1998). The application of an advanced ion exchange process to reprocessing spent nuclear fuels, (I)—Separation behavior of fission products from uranium. J. Nucl. Sci. Technol..

[43] Katsoyiannis I.A., Althoff H.W., Bartel H., Jekel M. (2006). The effect of groundwater composition on uranium(VI) sorption onto bacteriogenic iron oxides. Water Res..

[44] Barton C.S., Stewart D.I., Morris K.S., Bryant D.E. (2004). Performance of three resin-based materials for treating uranium-contaminated groundwater within a PRB. J. Hazard. Mater..

[45] Zhang S.-C., Ning S.-Y., Zhou J., Wang S.-Y., Zhang W., Wang X.-P., Wei Y.-Z. (2020). New insight into the adsorption of ruthenium, rhodium, and palladium from nitric acid solution by a silica-polymer adsorbent. Nucl. Sci. Tech..

[46] Chen X.N., Wang X.H., Fang D. (2020). A review on C1s XPS-spectra for some kinds of carbon materials. Fuller. Nanotub. Carbon Nanostructures.

[47] Greczynski G., Hultman L. (2021). The same chemical state of carbon gives rise to two peaks in X-ray photoelectron spectroscopy. Sci. Rep..

[48] Shchukarev A.V., Korolkov D.V. (2004). XPS study of group IA carbonates. Cent. Eur. J. Chem..

[49] Smith M., Scudiero L., Espinal J., McEwen J.S., Garcia-Perez M. (2016). Improving the deconvolution and interpretation of XPS spectra from chars by ab initio calculations. Carbon.

[50] Idriss H. (2021). On the wrong assignment of the XPS O1s signal at 531-532 eV attributed to oxygen vacancies in photo- and electro-catalysts for water splitting and other materials applications. Surf. Sci..

[51] He X.X., Tang J., Wang Z., Feng W.N., Yan Q.Q., Wei Y.Z., Watabe H., Li W.L., Ning S.Y., Chen L.F. (2024). Method and mechanism for efficient radium isolation from bulk thorium based on anion exchange. Chem. Eng. J..

[52] Dolatyari L., Yaftian M.R., Rostamnia S. (2017). Th(IV)/U(VI) Sorption on Modified SBA-15 Mesoporous Materials in Fixed-Bed Column. Iran. J. Chem. Chem. Eng.-Int. Engl. Ed..

[53] Dimiropoulos V., Katsoyiannis I.A., Zouboulis A.I., Noli F., Simeonidis K., Mitrakas M. (2015). Enhanced U(VI) removal from drinking water by nanostructured binary Fe/Mn oxy-hydroxides. J. Water Process Eng..

[54] Jamali M.R., Assadi Y., Shemirani F., Hosseini M.R.M., Kozani R.R., Masteri-Farahani M., Salavati-Niasari M. (2006). Synthesis of salicylaldehyde-modified mesoporous silica and its application as a new sorbent for separation, preconcentration and determination of uranium by inductively coupled plasma atomic emission spectrometry. Anal. Chim. Acta.

[55] Wang L., Song H., Yuan L., Li Z., Zhang Y., Gibson J.K., Zheng L., Chai Z., Shi W. (2018). Efficient U(VI) Reduction and Sequestration by Ti_2_CT_x_ MXene. Environ. Sci. Technol..

[56] Liu H.J., Zhou Y.C., Yang Y.B., Zou K., Wu R.J., Xia K., Xie S.B. (2019). Synthesis of polyethylenimine/graphene oxide for the adsorption of U(VI) from aqueous solution. Appl. Surf. Sci..

