# Supplementary material for: Efficient Uranium Removal from Aqueous Solutions Using Silica-Based Adsorbents Functionalized with Various Polyamines
Source: Toxics. 2024 Sep 27;12(10):704. doi: 10.3390/toxics12100704 (PMC11510947; doi:10.3390/toxics12100704)
Supplement: Supplementary file 1 [file toxics-12-00704-s001.zip › toxics-3212375-supplementary.pdf]

Efficient uranium removal from aqueous solutions using silica-based adsorbents functionalized with various polyamines

Ping Zhang [a], *Hongling Wang*[b,*], *Lifeng Chen* [c], *Wenlong Li* [c], *Toyohisa Fujita* [a], *Shunyan Ning* [c], *Yuezhou Wei* [c,d]

[a] State Key Laboratory of Featured Metal Materials and Life-cycle Safety for Composite Structures, School of Resources, Environment and Materials, Guangxi University, Nanning 530004, P.R. China.

[b] Institute of Resources Utilization and Rare Earth Development, Guangdong Academy of Sciences, 363 Changxing Road, Guangzhou 510650, P.R. China.

[c] School of Nuclear Science and Technology, University of South China, 28 Changsheng West Road, Hengyang 421001, P.R. China.

[d] School of Nuclear Science and Engineering, Shanghai Jiao Tong University, 800 Dong Chuan Road, Shanghai 200240, P.R. China.

*Corresponding author: Hongling Wang, E-mail: wanghongling@grre.gd.cn

S1. Synthesis of SiPMANs

Step 1: SiPMA preparation

First, 100 g of SiO_2 particles was placed in the glass flask of a rotary evaporator (EYELA N-300V-WB, Japan). The evaporator was then evacuated using a vacuum pump for ~30 min to ensure air-tightness. Next, 2.39 mL of DVB (monomer), 20.62 mL of MA (monomer), 45.67 mL of acetophenone (diluent), 30.45 mL of diethyl phthalate (diluent), and 0.3262 g of AIBN (initiators) were prepared and mixed homogeneously. The mixture was drawn into the flask under vacuum. The flask was then rotated continuously to ensure that the organic phase fully impregnated the pores of SiO_2. Once SiO_2 no longer adhered to the surface of the flask, rotation was continued for an additional 30 min. Nitrogen gas (N_2) was then introduced into the flask to protect the reaction, and the air pressure was maintained at 950 hPa. The flask was finally heated in water for 1 h at 60°C, 2 h at 70°C, and 8 h at 80°C. Once the reaction was complete, SiPMA was alternately washed with acetone and UPW and then dried at 45°C in a drying oven.

Step 2: Grafting of the polyamine functional groups

First, 10 g of SiPMA and 25 mL of acetophenone were poured into a 100 mL three-necked flask. SiPMA was swollen at room temperature with stirring at 70 rpm for 30 min. Then, 40 mL of TEPA was added to the flask. The agitation speed was adjusted to 90 rpm, and the reaction was conducted at 130°C for 13 h. After the reaction, the product was filtered through a sand funnel and washed alternately with ethanol and deionized water three times. The wet product was vacuum-dried at 45°C for 48 h.

Finally, a pale yellow powder was obtained and named SiPMA-TEPA. The preparation of SiPMA-DETA and SiPMA-TETA followed the same procedure as that of SiPMA-TEPA, except that the amine agents DETA and TETA were added at 23 mL and 31.27 mL, respectively.

S2. Batch experiments

(1) Experiments on the effect of pH

First, 0.05 g of SiPMAN was weighed and mixed with 30 mL of a working solution containing 1.0 ppm U and 3.0 mM $NaHCO_3$. The pH of the working solution was adjusted to 4, 5, 6, 7, 8, 9, and 10 through the addition of 1 M HNO_3 and 1 M NaOH. The samples were placed in a water bath shaker for 2 h, with the temperature and shaking speed controlled at 25°C and 120 rpm, respectively.

(2) Experiments on adsorption kinetics

First, 0.05 g of SiPMAN resin was weighed and mixed with a 30 mL working solution containing 10.0 ppm U and 3.0 mM $NaHCO_3$. The pH of the working solution was adjusted to 4, 5, 6, 7, 8, 9, and 10 using 1 M HNO_3 and 1 M NaOH. The contact times were controlled at 3, 5, 10, 20, 40, 60, 100, and 120 min. The samples were placed in a water bath shaker for 2 h at 25 °C with a shaking speed of 120 rpm.

(3) Experiments on the effects of major interfering ions

First, 0.05 g of SiPMAN resin was weighed and mixed with 30 mL of a working solution containing 10.0 ppm U and 3.0 mM $NaHCO_3$; NaCl was added to the working solution to achieve final concentrations of 0, 5, 10, 20, 40, or 100 mM. The

above steps were repeated for KNO_3 and Na_2SO_4. The pH of the working solution was adjusted to 7.0 using 1 M HNO_3 and 1 M NaOH. The samples were then placed in a water bath shaker for 2 h at 25°C, with the shaking speed set to 120 rpm.

In addition to the above interfering ions, a large number of calcium and magnesium ions are also present in the environmental water systems, and their effects on the adsorption efficiency need to be examined.

First, 0.05 g of SiPMAN resin was weighed and mixed with 30 mL of a working solution containing 1.0 ppm U and 3.0 mM $NaHCO_3$; $CaCl_2$ was added to the working solution to achieve final concentrations of 0, 0.5 or 5 mM. The above steps were repeated for $MgCl_2$. The pH of the working solution was adjusted to 7.0 using 1 M HNO_3 and 1 M NaOH. The samples were then placed in a water bath shaker for 2 h at 25°C, with the shaking speed set to 120 rpm.

(4) Experiments on desorbent selection and desorption kinetics

First, 0.05 g of SiPMAN resin was weighed and mixed with 10 mL of a working solution containing 1.0 ppm U and 3.0 mM $NaHCO_3$. The pH of the working solution was adjusted to 7 using 1 M HNO_3 and 1 M NaOH. After 2 h, the resin and solution were filtered through a sand core funnel, and the wet resin was dried in a vacuum oven at 45 °C for 48 h. After drying, the SiPMA-TEPA-U resin was desorbed using 10 mL of 0, 0.001, 0.01, and 1 M $HN0_3$. To study the desorption kinetics, 1 M HNO_3 was used as the desorbent, and the desorption times were set at 3, 10, 20, 30, 40, and 60 min.

(5) Experiments on uranium removal performance in environmental water systems

First, 0.05 g of SiPMA-TEPA resin was weighed and mixed with 30 mL of a working solution containing 1.0 ppm U. Both UPW and simulated groundwater were spiked with 3.0 mM $NaHCO_3$. The simulated groundwater also contained 40 ppm of calcium and magnesium ions. The working solutions of TW, RW, and WW were maintained at their original pH levels. These samples were placed in a water bath shaker, with the temperature and shaking speed controlled at 25°C and 120 rpm, respectively.

(6) Experiments on the adsorption isotherm of SiPMA-TEPA

First, 0.05 g of SiPMAN resin was weighed and mixed with 30 mL of a working solution containing 3.0 mM $NaHCO_3$ and varying concentrations of uranium at 10.0, 20.0, 50.0, 100.0, 200.0, or 300.0 ppm. The pH of the working solution was adjusted to 6 using 1 M HNO_3 and 1 M NaOH. The samples were then placed in a water bath shaker for 2 h, with the temperature and shaking speed controlled at 25°C and 120 rpm, respectively. The environmental water samples were collected from the WW at Yumu Mountain in Hengyang.

S3. Introduction of the mathematical models used

(1) The pseudo-first-order kinetic model (PFO) (Eq. (S1)) and pseudo-second-order kinetic model (PSO) (Eq. (S2)) were used to confirm the rate controlling step and investigate the adsorption mechanism.

PFO: $$Q_t = Q_e(1-e^{-k_1 t}) \quad \text{(S1)}$$

PSO: $$Q_t = \frac{Q_e^2 K_2 t}{1 + Q_e K_2 t} \quad \text{(S2)}$$

where Q_t and Q_e denote the adsorbed amount of the adsorbent at time t and at adsorption equilibrium, respectively. k_1 and k_2 denote the pseudo-first-order kinetic model and pseudo-second-order kinetic model rate constants, respectively.

(2) The Langmuir isotherm model (Eq. (S3)) assumes that the active sites on the surface of the adsorbent are homogeneous, that all active sites possess the same adsorption energy, and that the adsorbate is arranged in a monomolecular layer on the surface of the adsorbent. Additionally, the Freundlich isotherm model (Eq. (S4)) is an empirical mathematical model that assumes that the adsorption process is based on multilayer adsorption.

Langmuir isotherm model: $$Q_e = \frac{q_m K_L C_e}{1 + K_L C_e} \quad \text{(S3)}$$

Freundlich isotherm model: $$Q_e = K_F C_e^{\frac{1}{n}} \quad \text{(S4)}$$

where Q_e (mg/g) and q_m (mg/g) are equilibrium adsorption capacity and calculated saturation adsorption capacity; C_e (mg/L) refers to equilibrium ion concentration; K_L (L/mg) and K_F ($mg^{1-n}{\cdot}L^{n/g}$) are constants of the Langmuir and Freundlich isotherm models, respectively; and n refers to the adsorption intensity.

S4. Experiments on the reusability of SiPMA-TEPA

First, 0.05 g of SiPMAN resin was weighed and mixed with 10 mL of a working solution containing 10.0 ppm U and 3.0 mM $NaHCO_3$. The pH of the working solution was adjusted to 7 using 1 M HNO_3 and 1 M NaOH. After the adsorption

reaction, the solution was filtered through a sand core funnel, and the wet resin was dried in a vacuum oven at 45°C for 48 h. Following drying, the resin (SiPMA-TEPA-U) was desorbed using 10 mL of 1 M HNO_3. The solution from the desorption reaction was filtered through a sand core funnel and dried in a vacuum oven at 45°C for 48 h, The dried resin was then used for the next adsorption–desorption cycle. The SiPMA-TEPA resin was evaluated over five cycles.

S5. Sample preparation for the adsorption mechanism investigation

Owing to the size limitations of laboratory glass vials, a high concentration of uranium was used in the adsorption process to ensure that the SiPMA-TEPA resin was fully saturated. Specifically, 0.5 g of SiPMA-TEPA resin was weighed and mixed with 30 mL of a working solution containing 1000.0 ppm U and 3.0 $NaHCO_3$. Because highly enriched uranium tends to precipitate under neutral or weakly alkaline conditions, the pH of the working solution was adjusted to 6 using 1 M HNO_3 and 1 M NaOH.

S6. Experimental results

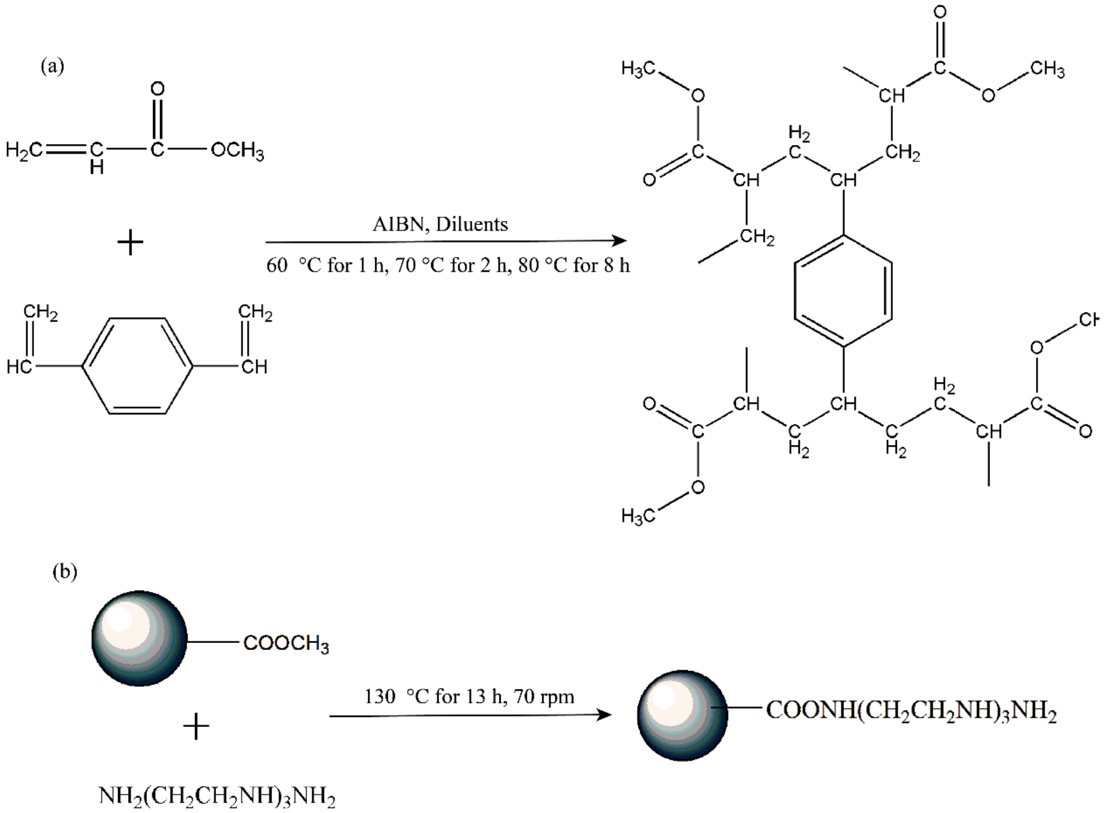

Figure S1. MA-and-DVB-crosslinked copolymerization (a) and substitution reaction between SiPMA and TEPA (b).

Table S1. Elemental contents of SiPMAN resins

Samples	N (%)	C (%)	H (%)
SiPMA-DETA	4.03	11.79	2.025
SiPMA-DETA	4.13	11.65	1.931
SiPMA-TETA	3.78	12.04	2.044
SiPMA-TETA	3.65	12.16	2.150
SiPMA-TEPA	4.21	12.92	2.229
SiPMA-TEPA	4.30	13.07	2.341

Table S2. Parameters obtained by fitting adsorption isotherm data for different solutions

Solution	Langmuir model			Freundlich model			$Q_{e,\,exp}$ (mg/g)
	K_L (L/mg)	q_m (mg/g)	R^2	K_F ($mg^{1-n} \cdot L^{n/g}$)	q_m (mg/g)	R^2	
Ultrapure water	1.92E−09	302.12	0.998	2.88	311.51	0.997	299.16
Groundwater	6.27E−09	285.36	0.962	1.38	304.10	0.940	270.00

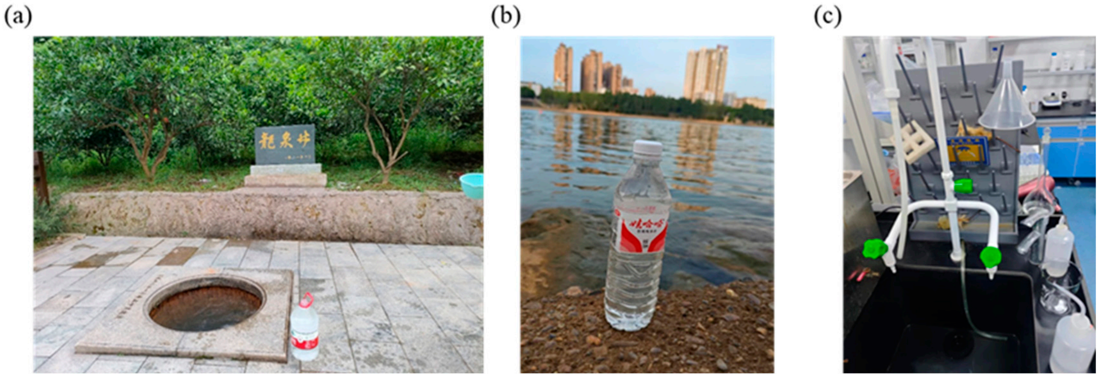

Figure S2. Well water of Mt. Yumu, Hengyang City (a); water collected from Xiangjiang River, Jiefang Road, Hengyang City (b); and tap water from Science and Innovation Building 406, University of South China (c).

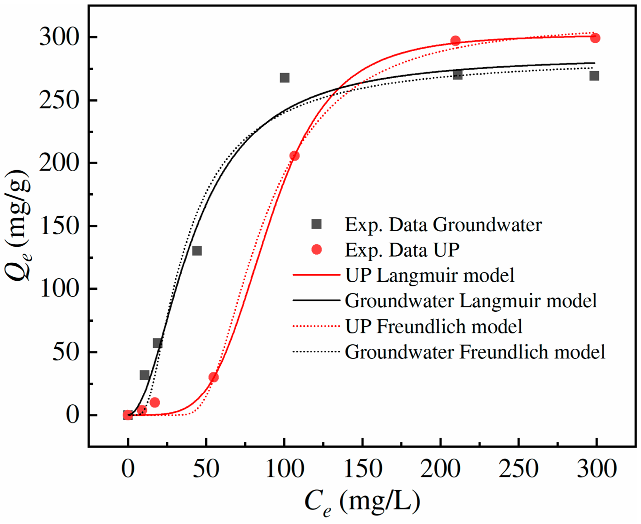

Figure S3. Adsorption isotherms for uranium removal by SiPMA-TEPA in ultrapure water (UPW) and groundwater (temperature 298 K, t: 2 h, pH = 6, *m*/*v*: 0.01 g/30 mL).

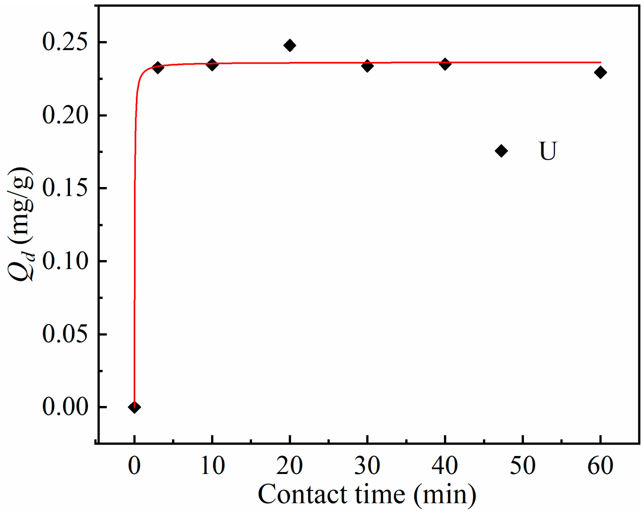

Figure S4. Desorption kinetics.
